# Older persons’ existential loneliness, as interpreted by their significant others - an interview study

**DOI:** 10.1186/s12877-017-0533-1

**Published:** 2017-07-10

**Authors:** Helena Larsson, Margareta Rämgård, Ingrid Bolmsjö

**Affiliations:** 1 Faculty of Health and Society, Department of Care Science, Malmö University, SE 205 06 Malmö, Sweden; 2 Department of Health and Society, Kristianstad University, SE 291 88 Kristianstad, Sweden

**Keywords:** Existential loneliness, Significant other, Older person, Qualitative, Interview study, Content analysis

## Abstract

**Background:**

In order to better understand people in demanding medical situations, an awareness of existential concerns is important. Studies performed over the last twenty years conclude that when dying and death come closer, as in the case with older people who are stricken by infirmity and diseases, existential concerns will come to the fore. However, studies concerning experiences of existential loneliness (EL) are sparse and, in addition, there is no clear definition of EL. EL is described as a complex phenomenon and referred to as a condition of life, an experience, and a process of inner growth. Listening to someone who knows the older person well, as significant others often do, may be one way of learning more about EL.

**Methods:**

This study is part of a larger research project on EL, the LONE study, where EL is explored through interviews with frail older people, their significant others and health care professionals. The aim of this study was to explore frail older (>75) persons’ EL, as interpreted by their significant others. The study is qualitative and based on eighteen narrative interviews with nineteen significant others of older persons. The data was analysed using Hsieh and Shannon’s conventional content analysis.

**Results:**

According to the interpretation of significant others, the older persons experience EL (1) when they are increasingly limited in body and space, (2) when they are in a process of disconnecting, and (3) when they are disconnected from the outside world.

**Conclusion:**

The result can be understood as if the frail older person is in a process of letting go of life. This process involves the body, in that the older person is increasingly limited in his/her physical abilities. The older person’s long-term relationships are gradually lost, and finally the process entails the older person’s increasingly withdrawing into him- or herself and turning off the outside world. The result of this study is consistent with previous research that has shown that EL is a complex phenomenon, but the implications of this research include a deepened understanding of EL. In addition, the study highlights the interpretations of significant others.

**Electronic supplementary material:**

The online version of this article (doi:10.1186/s12877-017-0533-1) contains supplementary material, which is available to authorized users.

## Background

Globally, with advances in medicine helping more people to live longer lives, the number of people over the age of 60 is expected to double within the next thirty years and reach two billion [1]. This demands radical societal changes, especially in order to ensure well-being, meaningfulness, and dignity for older people [2]. Furthermore, in order to better understand people in demanding medical situations, an awareness of existential concerns is important. Studies performed over the last twenty years conclude that when dying and death come closer, as in the case with older people who are stricken by infirmity and diseases, existential concerns will come to the fore [3–5]. In the health care context, the professionals need to be prepared to take care of the different needs of patients’, as well as of their significant others’, and not merely medical problems but also psychological and existential needs should be in focus [6]. However, studies show that professionals see existential concerns as a challenge [7, 8]. Studies also point out that the health care system fails to involve and take advantage of significant others’ knowledge and perception of the older persons’ situation [9, 10]. Thus, in order to provide adequate care and obtain a better understanding of how to meet older people in situations where existential needs appear, additional research on existential concerns is called for.

One existential issue in human life is existential loneliness (EL). A literature review by Mayers and Svartberg describes EL as a basic sense of loneliness that may occur when we, as human beings, face the fact that we are all alone in the world despite having other people around, but the review concludes, that the experiences of EL are unclear [11]. Another review views EL as a concept that consists of three dimensions: EL as a fundamental condition of human existence since we, as human beings, are separated from others; EL as an experience of loneliness without any relatedness to other people, described as feelings of nothingness and emptiness; and EL as a process of inner growth in which the negative experience of humans’ lonely nature is transformed into something positive [12]. Further, the psychotherapist Yalom, who has explored existential concerns in human beings’ lives, has referred to EL as a stipulation of life and pointed to how life itself involves EL. He writes about what he calls “the ultimate concerns”, such as the inevitability of death, our need of freedom and belongingness, and our search for meaning [13], which are all connected to EL. A number of philosophers have also written about the existential dimension of loneliness [14, 15]. Paul Tillich, for example, writes that being a human is to exist in a body that is alone and isolated, separated from everyone else’s [14]. Victor Frankl points out that the existential dimension of loneliness is never something that we can ignore, since it is part of being a human [15]. In addition, EL has been mentioned in qualitative studies within the health care context, especially when dying and death come closer [16, 17]. According to the literature referred to above, EL is a phenomenon that does exists, but despite the fact that philosophers and researchers have tried to clarify EL, there is still an indistinctness with regard to the experiences of EL and how EL is communicated. Thus, empirical studies would be helpful in understanding the phenomenon.

Listening to the voices of significant others may be one way of learning more about EL. It is well known that significant others play a crucial role for older persons’ well-being [10, 18], and they might be the ones who are closest to the older person and the ones who can provide invaluable information about the older person’s situation [19]. Qualitative studies describe the multifaceted role of being a significant other of older persons in need of care [20, 21], and this role is exhausting not only physically but also psychologically [22]. However, research also points to the fact that significant others want to take part in the care, and feelings of joy and satisfaction are described when significant others feel that they are counted on and listened to [19, 23]. Despite this, research reveals that significant others are often excluded when they should instead be seen as an asset [10, 24] and as a resource, that is, as experts and as companions in the care of older persons [9, 24, 25]. The studies performed during the last ten years, referred to above, concerning the experiences of significant others’, have primarily focused on how significant others perceive the care the older persons receive in their home or in hospital, or on how they themselves, as significant others, experience the situation of being close to someone who is aging. So far, there are few studies with a focus on how significant others notice and perceive existential phenomena experienced by their aging relative or friend, despite the fact that researchers and professionals point out the importance of listening to the voices of significant others in order to better understand older peoples’ needs. Early detection of existential suffering among frail older people is essential in order to prevent impaired existential health [4, 26] and in this endeavour the voices of significant others could be a valuable resource. Listening to someone who knows a person well [19], as significant others often do, might be helpful in identifying existential needs at an early stage.

Studies concerning experiences of EL are sparse and, in addition, there is no clear definition of EL [11, 12]. EL is described as a complex phenomenon [12], and, hence, multiple approaches are called for in order to explore and clarify the phenomenon. Therefore, empirical studies in general are needed, and in particular studies exploring the experiences of older people living in the nearness of death. Furthermore, exploring other kinds of views, such as those of significant others, might also generate a deeper understanding of the phenomenon. Hence, the aim of this study was to explore frail older (>75) persons’ EL, as interpreted by their significant others. This study is part of a larger research project on EL, the LONE study [27], where EL is explored through interviews not only with significant others but also with frail older people and health care professionals. The LONE study is in the development phase of designing a complex intervention [28].

## Methods

Since this study aims to explore human experiences, it is descriptive with a qualitative design [29]. The data was analysed using Hsieh and Shannon’s approach to conventional content analysis which allows interpretation of the content of data through a process of coding and categorising [30]. Hsieh and Shannon’s approach was chosen since their method is often used in a health care context, and since it is well described as well as useful in structuring a great amount of data. Moreover, this type of design and method for analysis seemed appropriate, since existing knowledge regarding EL is limited and since the aim was to explore a phenomenon as perceived through someone else’s eyes, rather than as experienced first hand [29, 30].

### Participants

The concept “frail older people” was defined as older persons >75 in need of long-term care related to multiple diagnoses, physical disorders, and functional impairments. The care was given by formal caregivers from the municipality or the county council. We hypothesised that older persons, who are stricken by infirmity and diseases, and who are likely to have suffered many different losses since they have lived for a long time, have experienced EL, and therefore we chose to ask them, their significant others and health care professionals about EL. Through interviews with frail older people (*n* = 23) (for a description of the care contexts, age, and gender, see Table 1), carried out by another researcher, the informants for this study were identified. The older persons were identified by a designated contact person at each care unit who provided oral and written information about the study. If the older person gave his/her allowance his/her name was communicated to one of the co-workers in the LONE study who then contacted the older person with the question to participate in the study. Findings from the interviews with the older persons are presented elsewhere [31]. The older persons were asked, after their participation in an interview, if they had anyone that was close to them and that might agree to be interviewed. Their answers resulted in a list of 20 persons and 19 of them were interviewed (for a description of the sample, see Table 2). For this study, we chose to use the concept “significant other” to designate the informants. It was the older persons who identified whom they wanted us to interview, and regardless of whom they named – friend, wife, daughter, etc. – the intrinsic meaning was the same, namely, a person who was close to them. In the following results, all the informants are referred to as “significant others.” If the older person gave the name of a significant other, that name was communicated (together with a telephone number) to HL (the first author). The significant other was contacted by telephone by HL, who inquired whether s/he would accept to receive a letter with information about the study. The letter contained information about the ethical aspects of the study and a description of EL, referred to as a deeper sense of loneliness. A couple of days after the letter had been sent out, the significant other was contacted again by telephone and asked whether s/he wanted to participate. If the significant other agreed to participate, s/he chose the time and the place for the interview. All the persons contacted, except one, wanted to participate.

**Table 1:** Description of the older persons’ care contexts, age, and gender

Older persons	*n* = 23
Men/women	12/11
Age, median (range)	85 (76–101)
Care context	*n* = 6
Primary health care centre	2
Municipal home care	4
Residential care	8
Hospital	2
Specialised palliative home care	6
Specialised palliative ward	1

**Table 2:** Description of the sample

Informants	*n* = 19
Men/women	6/13
Age, median (range)	63 (49–86)
Sons/daughters	5/4
Brother	1
Friend	1
Daughter-in-law	1
Wives	4
Cousin’s wife	1
Nieces	2

### Procedure

In eighteen interviews, nineteen significant others were interviewed. The interviews were individual, except one which was done with a couple. The informants received oral and written information about the aim of the study and about the procedure, together with a guarantee of confidentiality. They all gave both written and oral consent to participate. The interviewees all characterised their relation to the older person as close or very close. None of them were primary carers. The nature of the interviews was narrative. They followed a semi-structured guide with open-ended questions (see Additional file 1, for the Interview Guide), and were recorded digitally and then transcribed verbatim. The interviews lasted between 40 and 90 min (median = 51 min) and were conducted from February 2015 until August 2016. All interviews were done by HL, for the main part of the interviews with a senior researcher at her side. The interviews began by HL asking the informants to say something about the older person’s situation. Then the informants were introduced to the concept of EL as follows: “We are, in particular, interested in a deeper feeling of being alone in life, sometimes referred to as existential loneliness, a feeling that can come and go. Can you try to remember any situation when you perceived that your … experienced this kind of loneliness, this deeper feeling of being alone?” Follow-up questions were used when something came up that we thought was related to EL, questions such as “How did you notice EL?”, “Could you please tell me a little bit more about that?”, and “How would you put that feeling into words?”

### Data analysis

Hsieh and Shannon [30] describe three approaches to content analysis. One of those approaches, the one chosen for this study, is referred to as conventional and is characterised by staying close to the text in order to get familiarised with it and let categories emerge directly from the data [30]. Thus, firstly, all transcribed material was read and reread to get a sense of the text, and when something came up related to the aim, this was noted. The notes were written down and led to two further questions: “What kind of situations seem to trigger EL?” and “In what ways is EL expressed?” Secondly, the transcribed material was gone through word by word with those two questions in mind, and meaning units were identified related to the aim. Thirdly, each meaning unit was condensed and given a code, still close to the actual text. In the fourth step, the interpretation started and each code was given an interpretation. In the fifth and last step, the interpretations were brought together in clusters based on how they were related to each other. This step resulted in three categories that answer the question “As interpreted by the significant others, what circumstances seem to give rise to EL among older persons?”: *Being limited in body and space, Being in a process of disconnecting,* and *Being disconnected from the outside world*. All of the steps in the analysis were performed at first separately by the three authors and then together, comparing codes and interpretations until an agreement was reached.

## Results

### Being limited in body and space

The category *Being limited in body and space* illustrates how, according to significant others, limitations in frail older peoples’ lives lead to dependence on others. The limitations that they refer to are physical impairments in body and decreasing mobility in space. The decreasing mobility in space makes it difficult or impossible for older people to choose or decide for themselves where to be. When physical changes make older people incapable of deciding over their own body, they become increasingly dependent on staff and significant others. According to the significant others, the lack of personal freedom gives rise to EL. The body becomes a barrier to freedom and independence. In the example below, a significant other describes certain physical impairments that trigger EL. The older person who previously had been a very strong and vigorous man is now dependent on others for help with his most intimate needs.

*He says like, ‘if I could just use my hand’, he says, ‘that’s the worst of it all, this hand’//…and the incontinence and that he can’t…it’s the worst thing he knows. To have to rely on other people and to use diapers and all that, ugh, he thinks it’s horrible…he can’t do anything…//he says, ‘I’m totally worthless’…he also says that he’s pitiful…//…he wants to manage by himself…but he can’t* (21a)

According to significant others, the limitations of the body also affect the older person’s self-image. The dependence on others, as well as their inability to choose for themselves, give rise to certain feelings that older people have difficulties expressing to their significant others. The significant others refer to narratives where older persons say that they “see themselves as worthless,” and they also refer to situations where older persons use degrading words about themselves and show frustration. The significant others interpret these expressions of worthlessness and frustration as manifestations of EL due to older people’s increasing physical impairments.

*…it wasn’t funny many times…to hear ‘I don’t want to live anymore’, that ‘this isn’t a life worth living’…the impact of diseases and things like that, that she didn’t have the ability to move and participate any longer, it’s made her frustrated…//…we’ve always been doing things together, she’s been with us skiing in Austria and swimming in Germany and Belgium and we’ve been bicycling in the forest…and always been very active and all of a sudden not being able to move and be with us and be active…//…all of her life, now it’s not possible any more, ‘now I can hardly move, it’s not a life worth living’…//* (8a)

According to significant others, reduced mobility in space sometimes means lack of freedom. They perceive that EL arises when older persons can no longer decide in what place they want to be, and when they no longer have the ability to move between different environments. One example of this is an older person who earlier in life travelled a lot and through life has had different kinds of jobs which took him to many different places. As a disabled man he can still have some freedom driving around in his motorised wheelchair, but when he cannot use his motorised wheelchair any more he becomes dependent on other people and his personal freedom decreases because of his limitations in space.

*…he loves his motorised wheelchair and it’s been a feeling of freedom, he’s been driving out, down to the harbour, and he’s bought ice cream and he’s been looking around for a while, and when he no longer has this ability you notice that he thinks it’s very sad…//…this feeling of freedom, to look around, get impressions and experiences, decide yourself, maybe have control over what you…you decide yourself, now I want to go out and you do that, not being dependent on someone else…//* (4a)

In summary, when older people are no longer able to move around and choose for themselves where to go, they become limited in their spatial freedom and this may lead to EL. Being limited in body and space thus seems to give rise to EL, as interpreted by significant others.

### Being in a process of disconnecting

The category *Being in a process of disconnecting* illustrates how, according to significant others, frail older persons are in a continuous process of losing other people, places, and material belongings that they are or have been attached to. The many losses make older peoples’ lives empty and this may give rise to EL. The continuity in the older persons’ relationship to friends is often broken and they find it difficult to fill the gap. Saddened by this, they often think about people who have been important to them through life and especially their long-term relationships. The broken ties have to do not only with the fact that their friends get old and die, but also with difficulties in maintaining a relationship. This leads to emptiness since there is no longer anyone to share life with. Such a loss of emotional ties also means losing a part of themselves, as the person or persons they have lost cannot be replaced. The quote below illustrates a significant other’s description of the older person’s many losses.

*…who* [an acquaintance] *also passed away, unfortunately//So the two of them…became friends and they met a lot…Then she died too…Sad. It’s, it’s sad, so now she hasn’t got anyone…not any real friend//…they are fading away//And yes…since dad died…she’s sad of course//…that’s…when…that’s when she was left alone…lonesome…they had…lived all of their life together…and…yes, I think that…that they needed each other…to somehow fill out their lives in some way…//* (22a)

Older persons’ EL also seems to relate to the loss of their connection to cherished material belongings. According to the significant others, it is an emotional process of anxiety and melancholy for the older persons to know that they will have to let go of objects from their past that have a special value for them, or to imagine that those objects will not have any importance for anyone else after their death. To let go of one’s attachment to material belongings means to lose a part of oneself, which may lead to EL. Significant others have the impression that older persons are aware that the end of life is approaching, and to talk about their belongings becomes a way to pass on the story of their life. One example is a significant other who describes how the older person wants to tell the story of her life by talking about the objects connected to her past that have been important for her.

*…she tells a lot about her life too, she’s very keen to somehow pass things on, she tells me//she’s keen for me to know that this tapestry, for example, was made by her aunt, I notice it, she…it troubles her a bit, I think, that when she’s gone, the things will be dispersed in a sale, you know, and no one knows about her memory-laden things. She talks a lot about those things…she anchors her history in some way//* (1b)

The older person’s life story can also be related to a certain place. As interpreted by significant others, the process of losing the connection to a certain place leads to EL. The significant others notice expressions of sadness and grief among older people who look back on certain places of importance that they no longer have contact with. One example of this is a significant other who recounts how an older person was unable to stay in his house in the countryside where he wanted to live and dreamt of living until the end.

*….he always gets…he’s very sad when he returns from there, always, always…//Because he wants to be there, he’s…well, he…we’ve lived in many places and he’s renovated houses here and there and apartments and he’s…but he’s never been so attached to anything before this place…//He thought we would live for the rest of our lives at that place…but it didn’t turn out like that//…there are many losses…that he thinks about//* (21a)

The many and continuous losses make EL surface among older people. When they are in the process of looking back on painful memories, this is especially evident. The significant others feel that the memories often have to do with things in life that the older person wishes had turned out differently, but that are now too late to change. These memories are often filled with regret over a choice in life that was wrong or with sadness that different choices were not possible to make. The significant others describe how they notice that this mood is painful for the older person and that it triggers EL and is expressed as regret and as an inability to feel inner peace.

*She hasn’t got…she hasn’t…she hasn’t got inner peace, she hasn’t lived the life she wished for, she’s lived a life where she’s been…well, in…not forced to, probably not, but the choices she’s made have led to a life that she didn’t choose…*(5b)

In summary, the significant others perceive that older persons are in the process of continuously losing their connection to other people, places, and material belongings that they are or have been attached to, and that they find it hard to fill the gap left by those losses. This process leads to an increasing emptiness and makes older people experience EL. Being in a process of disconnecting thus seems to give rise to EL, as interpreted by significant others.

### Being disconnected from the outside world

The category *Being disconnected from the outside world* illustrates how, according to their significant others, frail older persons no longer have a sense of being part of a community, but rather are in a state of alienation, which makes their lives lonesome and gives rise to feelings of meaninglessness. One illustration of this is shown in the quotation below, where a significant other tells of an older person, living at a nursing home, who feels no companionship in the context she lives in because the other people living at the same nursing home are not able to talk or connect with each other at the dinner table. The significant other describes how the older person’s life becomes disconnected and how this seems to trigger EL.

*…and I mean the meals…sure, it’s good that they are gathered, but I mean there are…I think there are eight residents…and I think that half of them have to be fed…and of course, to be ninety-five years old and have to confront this, it can’t be only positive//…she thinks there’s no one else…and there isn’t anyone else, more than her, at the unit who has a clear mind, as she does…//* (11a)

Significant others perceive that older persons have difficulties in communicating their disconnection, but that they try to find words that explain how they feel. Below, a significant other relates what an older person has said, which she understands as if the older person describes disconnection. The older person seems to voice EL by saying that the days have become quiet and silent.

*…because she says ‘well, yesterday was a silent day, yesterday was such a silent day’…but I say, so and so was supposed to come for a visit. ‘Well, they were here, but it was a very silent day’* (11a)

Several of the significant others feel that they can tell just by looking at the older person that s/he is disconnecting. According to significant others, the disconnection from the outside world is seen regardless of where the older person lives. Sometimes they feel that they have trouble connecting with the older person, who increasingly turns off the outside world and withdraws into him- or herself. The significant others, also point out that it is not always easy to understand the older person’s expressions of disconnection, and they recount how although the older persons do not always describe their situation with words they nevertheless do so with their body language. Below, a significant other describes how the older person shows with her body that she has turned off the outside world.

*…then she’s sitting like this, in this way* [hanging over the table]*, I usually say that it’s like a computer that stalls…that’s shutting down, and that’s how my mom sits* (14a)

Older persons often have difficulties putting their experiences of disconnection and meaninglessness into words. However, on hearing expressions like “There’s no meaning any more,” the significant others sometimes think that the older persons experience disconnection. The significant others understand these words as if the older persons feel that their lives have nothing more to give, and as a longing for life to end, that is, as a longing to die. In summary, when life becomes increasingly disconnected from the outside world and meaningless for older people, they experience EL. Feelings of being disconnected thus seem to give rise to EL, as interpreted by significant others.

## Discussion

The result of this study shows that the significant others perceive that the older persons experience EL (1) when they are increasingly limited in body and space, (2) when they are in a process of disconnecting, and (3) when they are disconnected from the outside world. The comprehensive understanding of the result is that the older person is in a process of letting go of life.

A key finding is that the significant others perceive that the older persons’ physical impairments lead to EL. There is plenty of research concerning professionals’ experiences of meeting older persons’ existential needs [7, 8, 16] but only one study that relates existential challenges to the physical body [16]. A study by Whitaker [32] describes older persons’ experiences of the body as a constant reminder of the finitude of life, and a similar result is shown in research related to palliative care [16]. The idea of the deteriorating body can be related to Gabriel Marcel, according to whom an existential situation can be described as a trial when we, in our body, confront time [33]. In the present study, this is illustrated by the decay of the physical body as experienced by older people. One experience in the trial that Marcel talks of could be EL. In order to notice, recognise, and be able to interpret the older person’s bodily expressions, it is of importance to know the person well [19], as significant others often do. However, studies have shown that the situation of significant others is complex [22, 24, 34] and that the health care system fails to take advantage of their knowledge of the older person’s situation [9, 10]. Thus, the knowledge that the significant others’ possess, regarding for example the older persons’ bodily expressions, may never reach the health care professionals. This study demonstrates that significant others are a valuable resource in the understanding of the experience of EL.

Another key finding is that the significant others perceive that the older persons experience EL when they increasingly lose long-term relationships, either through an inability to maintain contact or through bereavement. Often, the older persons’ ability to communicate in social situations is also reduced. The significant others notice this and perceive that, as a result, EL surfaces among older persons. This finding can be related to a study that highlights experiences of aphasia [17]. Nyström’s interview study [17] concludes that without a communicative relational anchor, EL will occur. Furthermore, the importance of relations is emphasised by Marcel, who writes that the meaning of life is about the meeting between you and me [33]. If we as human beings do not have, or are unable to maintain, relationships, the meaning of life will be lost. When older persons are no longer capable of maintaining social contacts, the health care professionals become a vicarious relational anchor in their existence. However, several of the significant others talk about the fact that the older persons’ health care contact is not enough to fulfil their need for a relational anchor. Martin Buber claims that it is of importance to have *I and you* relations in order for a fruitful meeting to occur [35]. Such relationships usually exist between the older person and a person they refer to as their significant other. An approach that may enable an *I and you* relation between professionals and the older person is person-centred care. During the last few years, person-centred care has been favoured in several health care settings of Western societies and its philosophy is to focus on what is important for the person that receives care. In order to know something about what is important for a person, it is essential to build a relationship over time, which requires a certain continuity [36]. Several studies show that one of the greatest challenges to person-centred care is the increased time pressure and stress [36, 37] that reduce the opportunities for building a relationship and that are difficult to reconcile with the older person’s existential needs, which require genuine presence [17]. However, encountering the older person and his/her significant others, being able to sit down and talk for a while in a supporting care environment where the personnel feel encouraged and supported by each other and by the organisation, are examples of conditions that make a person-centred philosophy flourish in elderly care [36]. This study shows that significant others feel that the older person can experience EL even in a social context, and that there is a need to further explore the impact of the absence of relational anchorage on EL in older persons.

Finally, yet another key finding is that significant others perceive that the older person is increasingly dependent on others. This finding can be related to a lack of autonomy, because the older persons’ ability to make choices and influence their situation is significantly reduced. Freedom of choice and freedom to influence one’s own life are important aspects of autonomy. Another aspect of autonomy is the ability to influence, and to interact with, one’s environment [38]. Studies dealing specifically with autonomy linked to older people are sparse. Thus, it is difficult to know how older people experience their situation of dependence. In a concept analysis, four Swedish researchers show that there is a discrepancy between what is said about how autonomy should be safeguarded and how patients experience autonomy [38]. There are, however, few studies describing freedom in the context of EL. In a few previous studies, the situation of older people with reduced opportunities to choose, and to influence their own lives, is described as if a limit in life is reached [16, 32], which is likely to involve experiences of limited freedom. The link between the lack of freedom and EL can be seen in relation to Frankl’s contention that freedom consists of the possibility to choose [15]. Thus, if we as humans do not experience the freedom to choose, life has reached a limit. The significant others describe how they notice that the older person increasingly turns off the outside world and withdraws into him- or herself. According to the present study, this can be understood as if the older person withdraws in an attempt to seek inner peace, that is, a kind of inner freedom, when outer freedom is no longer possible. Buber [35] describes this state as a process of thinking, which can be seen as each person’s inner conversation with him- or herself, and this inner freedom cannot be taken away from anyone.

In summary, the result of this study is consistent with previous research that has shown that EL is a complex phenomenon. In addition, this study highlights the interpretations of significant others and shows that their experiences are valuable to take into account in order to understand EL among frail older people. Nevertheless, research demonstrates that significant others are excluded [10, 24] when they should be seen as a resource in the care of older people [9, 24, 25].

There are some limitations to this study. First, the study took place within a context of a Western society where loneliness is seen as a sensitive topic to talk about [16] and we had only one informant with an origin other than Western. In addition, the fact that we interviewed significant others of frail older persons in need of long-term care might have given the first category, *Being limited in body and space*, a greater significance than it would have had if the informants had been significant others of older people in general. These limitations potentially reduce the transferability [39] of the findings. Furthermore, we had to take into consideration that the concept of EL is not clarified and is seen as a complex phenomenon. Therefore, we were two interviewers, one junior researcher and one senior researcher familiar with existential conversations, so that one of us could have the role of an observer, making notes and at the end of the interview adding some more questions, if needed. Finally, to guarantee the dependability [39] of this study it was important to minimise the risk of basing interpretations on preunderstandings, and therefore all three researchers took part in the analysis and the interpretations. Furthermore, in order for the interpretations of the researchers to be verifiable [39], the reader of this study should be able to follow every step, and to this end quotations are used to exemplify the three categories that emerged during the analysis process.

## Conclusion

The implications of this research include a deepened understanding of EL. The result can be understood as if the older person is in a process of letting go of life. This process involves the body, in that the older person is increasingly limited in his or her physical abilities. The older person’s long-term relationships are gradually lost, and finally the process results in the older person increasingly withdrawing into him- or herself and turning off the outside world. What we can say is that these findings seem to be central to the experience of EL among frail older persons, as interpreted by their significant others. However, what we cannot say anything about is whether the findings reflect the older persons’ actual experience. Therefore, comparing the older persons’ experience with the perception of their significant others’ is an important topic for further research.

## Additional file

Additional file 1: Interview Guide (developed for this study; not previously published). (DOCX 13 kb)

## Abbreviations

EL: Existential loneliness
HL: Helena Larsson
IB: Ingrid Bolmsjö
MR: Margareta Rämgård
