# Supplementary material for: Older persons’ existential loneliness, as interpreted by their significant others - an interview study
Source: BMC Geriatr. 2017 Jul 10;17:138. doi: 10.1186/s12877-017-0533-1 (PMC5502486; doi:10.1186/s12877-017-0533-1)
Supplement: Additional file 1: — Interview Guide (developed for this study; not previously published). (DOCX 13 kb) [file 12877_2017_533_MOESM1_ESM.docx]

**Interview Guide**

Our colleagues have interviewed older persons about existential loneliness, and, as you are aware, they have interviewed your relative. Now we are interested to know something about *your* perspective. In order for us to better understand your relative’s experiences, could you please start by telling us about his/her situation?

We are, in particular, interested in a deeper feeling of being alone in life, sometimes referred to as existential loneliness, a feeling that can come and go. Can you try to remember any situation when you perceived that your … experienced this kind of loneliness, this deeper feeling of being alone?

Probing questions:

How did you notice this?

Do you remember what you were thinking?

Could you please tell us more about this?

How would you put that feeling into words?

How did you handle the situation?

How did you handle your own feelings?

Have you experienced something similar yourself?

Can you talk with your … about this kind of loneliness? How?

Can you/do you want to share these experiences with someone else? How?

How does it feel right now?
